# Transient recovery dynamics of a predator–prey system under press and pulse disturbances

**DOI:** 10.1186/s12898-017-0123-2

**Published:** 2017-04-04

**Authors:** Canan Karakoç, Alexander Singer, Karin Johst, Hauke Harms, Antonis Chatzinotas

**Affiliations:** 1 Department of Environmental Microbiology, Helmholtz Centre for Environmental Research-UFZ, Permoserstraße 15, 04318 Leipzig, Germany; 2 Department of Ecological Modelling, Helmholtz Centre for Environmental Research-UFZ, Permoserstraße 15, 04318 Leipzig, Germany; 3 Centre for Integrative Biodiversity Research (iDiv) Halle-Jena-Leipzig, Deutscher Platz 5e, 04103 Leipzig, Germany; 4 Swedish Species Information Centre, Swedish University of Agricultural Sciences, P.O. Box 7007, 75007 Uppsala, Sweden

**Keywords:** Pulse disturbance, Press disturbance, Transient dynamics, Recovery, Trophic interactions, Protist, Bacteria, Predation, Prey release, Food web

## Abstract

**Background:**

Species recovery after disturbances depends on the strength and duration of disturbance, on the species traits and on the biotic interactions with other species. In order to understand these complex relationships, it is essential to understand mechanistically the transient dynamics of interacting species during and after disturbances. We combined microcosm experiments with simulation modelling and studied the transient recovery dynamics of a simple microbial food web under pulse and press disturbances and under different predator couplings to an alternative resource.

**Results:**

Our results reveal that although the disturbances affected predator and prey populations by the same mortality, predator populations suffered for a longer time. The resulting diminished predation stress caused a temporary phase of high prey population sizes (i.e. prey release) during and even after disturbances. Increasing duration and strength of disturbances significantly slowed down the recovery time of the predator prolonging the phase of prey release. However, the additional coupling of the predator to an alternative resource allowed the predator to recover faster after the disturbances thus shortening the phase of prey release.

**Conclusions:**

Our findings are not limited to the studied system and can be used to understand the dynamic response and recovery potential of many natural predator–prey or host–pathogen systems. They can be applied, for instance, in epidemiological and conservational contexts to regulate prey release or to avoid extinction risk of the top trophic levels under different types of disturbances.

**Electronic supplementary material:**

The online version of this article (doi:10.1186/s12898-017-0123-2) contains supplementary material, which is available to authorized users.

## Background

Disturbance is one of the key drivers of the dynamics and diversity of communities [1–3] and is defined as a discrete event in time killing or damaging individuals [4]. Disturbances occur in many natural systems with different strengths and durations. They are often classified as pulse disturbances (short-term events) or press disturbances (long-term events) depending on their duration in relation to the generation times of species [5, 6]. These different temporal patterns of disturbances are important for understanding the structural and functional community responses [7]. Press disturbances, for instance, can cause increasing variability in the relative abundances of species, whereas pulse disturbances can cause dramatic structural and functional shifts [8].

Besides the characteristics of the disturbance, the traits of the species and their biotic interactions are important determinants of community responses [9, 10]. However, the indirect impacts of disturbances caused by the biotic interactions are not well understood and are often overlooked. In particular, the trophic status in food webs plays a major role for the species response to disturbances [11–13]. Traits such as large body size, slow growth rate and low population size make top predators more vulnerable than other trophic levels. Studies have been shown that a reduced top-down control allowed prey outbreaks with cascading changes in ecosystem structure and function [14–16]. Similarly, in a microcosm study, increasing temperature led to increasing invasion success of a bacterial prey species due to the increased prey release from protozoan predation stress [17].

It is well known that long transient phases of population dynamics may occur in response to disturbances [18] and particularly strong or long-term disturbances may prolong these transient phases [19]. Among the ecological attributes known to affect transient recovery dynamics, the presence and availability of resources are particularly important [8]. It was previously hypothesized that the availability of alternative resources for the predator may increase the persistence of predator–prey systems [20]. Moreover, foraging behavior may be flexible and may change in disturbed environments [21]. Surprisingly, little is known about how the coupling of the predator to an alternative resource affects the recovery dynamics.

In this study, we combined microcosm experiments and modelling to investigate transient recovery dynamics of a simple microbial food web (consisting of predator, prey and a common resource). We exposed this system to disturbances, which we applied as increasing dilution rates. We contrasted two different disturbance regimes (i) a discrete and severe disturbance (pulse), and (ii) a long term and mild disturbance (press). We monitored the abundances of predator and prey before, during and after the disturbance.

In a second step we investigated using an ecological model the transient dynamics of both trophic levels under different disturbance strengths and durations beyond those applied in the experiments. In particular, we studied the consequences of the predator coupling to the alternative resource for the transient recovery dynamics. We found that disturbance strength and duration were decisive for the different transient recovery dynamics of the two trophic levels. In particular, we observed a slowed down recovery of the predator inducing a transient phase of prey release, i.e. temporarily high prey population sizes. Our results also revealed the importance of the predator coupling to an alternative resource which strongly impacted the recovery time of the predator and thus the length of the prey release phase.

## Experimental methods and model

### Origin and maintenance of stock cultures

The bacterium *E. coli* JM109 harboring a chromosomal green florescent protein (GFP) was used as prey organism. Using this strain allowed us to monitor *E. coli* in the food vacuoles of protists and facilitated controlling for contamination. A single clone grown on a lysogeny broth (LB) agar supplemented with 50 mg/ml kanamycin was used for establishing a pre-culture in liquid LB medium. Incubation was done in 50 ml medium in a 200 ml culture flask for 24 h on a closed rotating shaker at 25 °C. A low salt LB medium (1% tryptone, 0.5% yeast extract, 0.5% NaCl, 50 mg/ml kanamycin) was used for incubation of bacterial pre-cultures. Pre-cultures of the protist *Tetrahymena pyriformis* were established in proteose peptone yeast extract medium (1% proteose peptone, 0.15% yeast extract, 0.01 mM FeCl_3_) at 25 °C in an incubator without shaking. These pre-cultures were cultivated axenically (i.e. growth on only dissolved nutrients without any bacteria) to avoid transfer of unwanted bacteria to the experimental cultures. *Tetrahymena pyriformis* is able to grow as a bacterivore (i.e. predating on bacterial prey) or as an osmotrophy (via direct uptake of dissolved nutrients). Prior to the experiments, pre-cultures of protists were concentrated by centrifugation (1000*g*, 10 min) and washed with experimental media twice. Both bacteria and protist pre-cultures were enumerated and diluted to the experimental concentrations with the experimental media. Enumeration techniques and all starting concentrations are described below. The *E. coli* JM109 and *Tetrahymena pyriformis* strain that were used in this work have been deposited at the public culture collection of the Department of Environmental Microbiology at the Helmholtz Centre for Environmental Research-UFZ (http://www.ufz.de/index.php?en=37703).

### Experimental conditions

We used the above mentioned low-salt (in order to prevent salt damage on protists) LB medium during the experiments as the growth resource for the bacterial prey. The complex carbon source of the LB medium (i.e. yeast extract) served as an alternative resource for the predator. All experimental media were sterilized and filtered through a 0.2-µm pore sized filter. Experiments comprised 20 ml semi-continuous cultures in 50 ml sterile disposable culture flasks. Experimental cultures were always incubated at 25 °C for 24 h without shaking and all other environmental parameters were kept constant.

We found that a daily tenfold dilution prevented the collapse of the populations and resulted in an equilibrium state at which prey and predator coexist. This daily dilution went along with a replenishment of resources (i.e. LB medium) before they were depleted. It also reduced cell debris and excretion products and prevented oxygen depletion during the experiments. The remaining culture after each transfer was used for cell counts.

### Experimental design

Three different treatments were applied: undisturbed (control), press disturbance and pulse disturbance. All treatments were replicated three times. All treatments were imposed by diluting the cultures with fresh medium as described below.

#### Undisturbed control

All replicate microcosms started with equal cell numbers of *E. coli* (3.6 × 10^7^ cells ml^−1^) and *Tetrahymena pyriformis* (4.2 × 10^4^ cells ml^−1^). Each day 2 ml from the cultures were transferred into 18 ml of fresh medium and allowed to re-grow for 24 h following this tenfold dilution.

#### Press disturbance

After control communities reached the equilibrium dynamics, they were exposed to the press disturbance from day 22 to 32 in separate flasks. Press disturbance was imposed as 40-fold daily dilution (simulating 4 times increased dilution rate compared to the daily constant rate) for a period of 10 days.

#### Pulse disturbance

Communities that had reached equilibrium dynamics were exposed to the pulse disturbance treatment on day 15. Pulse disturbance was applied as a single 2500-fold dilution (simulating a 250 times increased dilution rate). Initial cell numbers were lower than in the press experiment (i.e. 4 × 10^6^ for bacteria and 4 × 10^3^ for protists), but started with a similar predator: prey ratio as in the other treatments.

### Sampling

A well-mixed 500 µl subsample was fixed with 0.2% Lugol’s iodine solution for quantifying protists. Subsamples were diluted if the cells were too many to be counted reliably. Fixed protist cells were counted under an inverted microscope (Olympus CKX41, Olympus America Inc., Melville, NY, USA) with a Sedgewick-Rafter counting chamber (Pyser-SGI Limited, Edenbridge, UK). An additional 15 ml subsample was filtered through a 20 µm mesh filter (CellTrics, Sysmex Partec, Kobe, Japan) to remove protist cells prior to counting bacteria with a Coulter Counter (Multisizer 3, Beckman Coulter, Brea, CA, USA). Cell numbers were recorded every day.

### Growth curves

Growth rates of prey and predator were determined by growing the organisms under the same experimental conditions for 24 h (i.e. without dilution). The triplicate cultures contained only prey, predator growing axenically without prey, and prey and predator together. Initial abundances of *E. coli* and *Tetrahymena* sp. were 4 × 10^6^ and 2500 cells ml^−1^ respectively. Samples were taken with sterile syringes at 12, 14, 16, 18, 20, 22, 24 h. Protists and bacteria were counted as described above.

### Modelling

We modelled the microcosm experiments as a time-discrete version of a Lotka–Volterra type predator–prey model [22]. Particularly, the model considers predator coupling to an alternative resource and the action of disturbances. Justified by experimentally determined growth curves (Additional file 1: Figure S1), we assumed a density limited prey population (*P*) and an exponentially growing predator (*C*).

1a $$P_{t + 1} = (1 - d_{t} )P_{t} + r_{P} P_{t} \left( {1 - \frac{{P_{t} }}{{K_{P} }}} \right) - c_{P} P_{t} C_{t}$$

1b $$C_{t + 1} = (1 - d_{t} )C_{t} + r_{C} C_{t} + c_{C} P_{t} C_{t}$$

where d_*t*_ is the dilution rate (applied once per 24 h), *r*_*P*_ is prey growth rate without predators, *K*_*P*_ is prey carrying capacity, *c*_*P*_ is the prey interaction coefficient describing how much prey is consumed per predator, *r*_*C*_ is the predator growth rate without prey and *c*_*C*_ is the predator interaction coefficient describing the consumption and conversion of prey to changes in *C* (Table 1). The model was implemented in R (version 3.1.3; [23]).

**Table 1:** Parameter description and parameter values for the Eqs. (1a and 1b)

Name	Description	Value
*d*_*t*_	Dilution rate	0.9 days^−1^
*r*_*P*_	Prey growth rate	0.094 (7.5 min)^−1^
*r*_*C*_	Predator growth rate	0.012 (7.5 min)^−1^
*K*_*P*_	Prey carrying capacity	4.9 × 10^8^ cells ml^−1^
*c*_*P*_	Prey interaction coefficient	3.5 × 10^−6^ cells^−1^ ml
*c*_*C*_	Predator interaction coefficient	1.4 × 10^−11^ cells^−1^ ml

Note that the parameter *r*_*C*_ is important as it implicitly describes the coupling of the predator to another resource additionally to the prey population. Positive *r*_*C*_ imply coupling to this resource allowing the predator population to grow even in absence of prey. However, the model ignores a potential resource competition among predator and prey. Resource competition is unlikely, due to regularly strong dilution every 24 h. Dilution reduces the potential for resource competition in two ways: it removes predator and prey cells (i.e. reduces the amount of resource consumers) and it additionally renews the resource.

The model describes *C* and *P* as cells ml^−1^ and is iterated at a time step of 7.5 min, leading to 192 iterations per day. Initial tests showed that the step size was sufficiently small to cover the experimental dynamics measured daily. Model results are displayed in daily time steps corresponding with experimental sampling times. For clarity, we left out the modelling time steps at a finer scale. Therefore, decline due to dilution and regrowth within the 24 h between dilutions are not visible.

To calibrate the model, initially we adjusted parameter values to the measured growth curves (see Additional file 1: Figure S1). Growth rates and prey carrying capacity were calibrated from the respective single species growth curves. Subsequently, interaction parameters were calibrated using the growth experiment with both species. We applied the Nelder–Mead optimization algorithm [24, 25] in R within reasonable wide parameter ranges. We then refined the parameter estimates by calibrating the model additionally to the control treatment. For this purpose, we applied a Latin hypercube approach on a narrow parameter space around the parameter estimates from growth curves. We then selected the parameter set that minimized the fourth power of the sum of relative distances to all cell counts in the control experiment. With the additional calibration to the control experiment we accounted for the possibility of uncontrolled changes in conditions between the separate growth and disturbance experiments.

### Evaluation of results

We used the standard metric Nash–Sutcliffe efficiency (E) to quantify the general model efficiency in predicting the experimental data. E ranges between 1.0 (perfect fit) and −∞. An E that is lower than 0 means that the mean value of the experimental data could be a better predictor than the model [26].

To specifically assess the differences between model and data during the first days after the start of the press or the occurrence of the pulse disturbance, we calculated the time of the species response to the disturbance by detectable abundance changes. Specifically, we defined “response time” as the time between the start of the disturbance and the day when species population size left the range of equilibrium sizes (they were calculated for the period from day 7 until disturbance start). Difference between the response times of the model and the data (average of replicates) is stated as “deviation time *D*_*T*_”. Deviations between the recovery times were calculated in the same manner as response time.

For the evaluation of prey release we calculated the covariance between prey and predator population sizes before, during and after disturbance. A negative covariance implies that prey population size strongly increases due to decreasing predator population size thus exhibiting prey release.

### Simulation experiments

We applied the calibrated model to simulate situations that would have been difficult to directly control in the experiment. In simulation experiments, we tested the impact of (1) the duration of press disturbance, which we varied between 2 and 12 days, (2) the strength of pulse disturbance (varying between 10 and 10^6^ on a 10-logarithmic scale, and (3) the strength of the predator coupling to the resource by varying parameter *r*_*C*_ in the range of 0.007–0.011. In these experiments, we particularly focused on speed of predator recovery, which we calculated in terms of “recovery time”. Note that all source codes used in this manuscript are available upon request.

## Results

### Experimental population dynamics

**Fig. 1:**
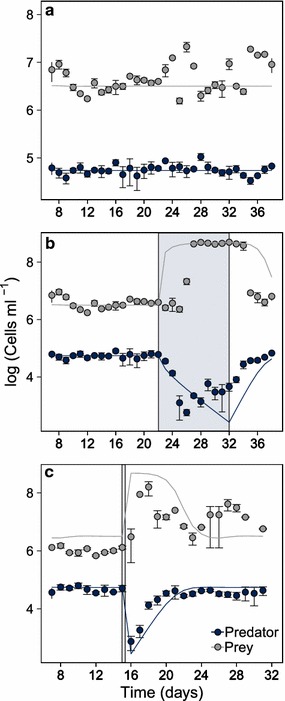
Transient dynamics of predator and prey without and with disturbances. *Grey* and *dark blue filled circles* correspond to the experimentally determined mean predator and prey population dynamics respectively. *Error bars* represent ± standard deviation. *Solid lines* correspond to the model simulations (only daily time steps are shown). **a** Population dynamics without disturbance; **b** under press disturbance and **c** under pulse disturbance. Control without disturbance is with tenfold daily dilution, press disturbance corresponds to 40-fold dilution between the days 22–32 and pulse disturbance to 2500-fold dilution on the day 15. Disturbance action is shown as *grey shadows*

In the control treatment, an equilibrium state appeared at which prey and predator coexisted (Fig. 1a). Under press disturbance, the prey population started to increase on day 26 reaching a higher equilibrium size than that of the control (Fig. 1b). At the end of the press disturbance, this high equilibrium population size remained for two more days and then turned back to the pre-disturbance size which was reached after full predator recovery on day 33 (at least two replicates were recovered). The predator population declined during press disturbance but started to increase during the disturbance period. After press disturbance ceased, the predator population recovered fully to its pre-disturbance size (Fig. 1b). Increasing negative covariance (*before disturbance cov* = −0.002*; during/after disturbance cov* = −0.292) indicated a phase of prey release during and after the disturbance (see “Evaluation of results”).

Under pulse disturbance, the prey population increased already after one day as a consequence of the reduced predator population size (Fig. 1c). The prey population did not return back to the pre-disturbance level by the end of the experiment. The predator population continued to decline after the pulse but started to recover soon to the pre-disturbance size within 3 days (at least 2 replicates were recovered). Increasing negative covariance (*before disturbance cov* = −0.003*; during/after disturbance cov* = −0.458) indicated a phase of prey release after the pulse disturbance (see “Evaluation of results”).

### Modeled population dynamics

As we calibrated our model to the control treatment without disturbance, the fitted model reproduced well the non-disturbed experimental data (Fig. 1a). Also the overall response patterns to the press and pulse disturbances were captured well by the model (Fig. 1b, c). Nevertheless, the modeled population dynamics showed some slight discrepancies to the experimental data. In the control treatment, predator dynamics (*E* = −0.01) were better predicted than the prey dynamics (*E* = −0.32). This is also true for the press disturbance (*E* = 0.34 *and E* = 0.11, respectively) and even more pronounced for the pulse disturbance (*E* = 0.61 *and E* = −0.44, respectively).

Specifically, some differences between modeled and experimental population dynamics occurred during the first days after the start (press) or the occurrence (pulse) of disturbance. Under press disturbance, the projected prey population showed an earlier response (*D*_*T*_ = −3) and late recovery (*D*_*T*_ > 4.3), (Fig. 1b). During the press disturbance, the experimental predator population started to increase already within the disturbance duration (around day 28), whereas the modelled population continuously declined, started to increase only after press disturbance ceased at day 32 (*D*_*T*_ = −1) and recovered later (*DT* = 4.6), (Fig. 1b).

Under pulse disturbance, the projected prey population size was slightly higher during the pre-disturbance and disturbance period. Experimental prey populations did not recover *until the end of the experiments* (see “Limitations and outlook”; Fig. 1c). Predator recovery to the equilibrium state was longer than in the experiments (*D*_*T*_ = 4.3).

**Fig. 2:**
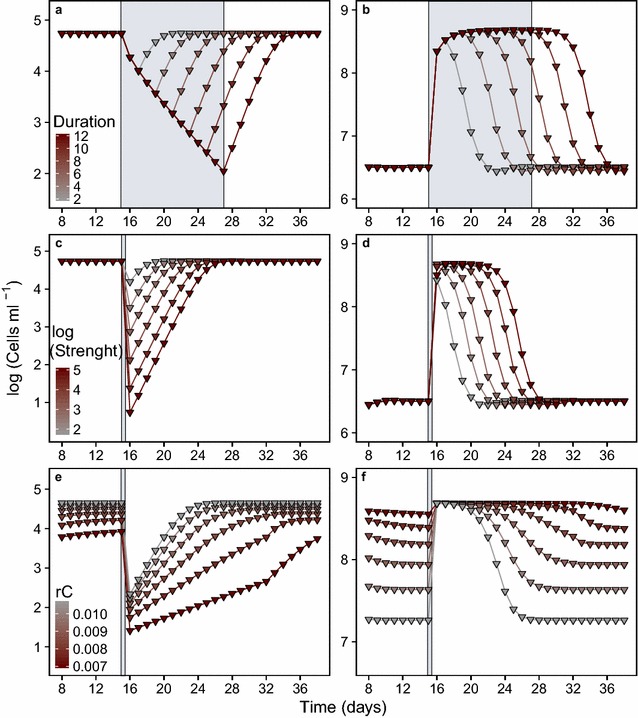
Impact of disturbance duration, strength and predator coupling to an alternative resource on transient dynamics. **a**, **b** Impact of press disturbance duration (varied from 2 to 12 days) projected by the model simulations for predator (**a**) and prey (**b**). Strength of disturbance was kept constant (40-fold). Disturbance has started on day 15. *Color gradient* shows the shortest (*grey*) to the longest (*dark red*) disturbance duration. **c**, **d** Impact of pulse disturbance strength (varied from 50 to 100,000-fold) projected by the model simulations for predator (**c**) and prey (**d**). *Color gradient* shows the lowest (*grey*) to the highest (*dark red*) disturbance strength. **e**, **f** Impact of predator coupling *r*_*C*_ to an alternative resource besides prey (varied from 0.007 to 0.011) under pulse disturbance (2500-fold dilution) projected by the model simulations for predator (**e**) and prey a (**f**). *Color gradient* shows the lowest (*grey*) to the highest (*dark red*) *r*_*C*_ values. Disturbance action is shown as *grey shadows*

Having found qualitatively similar community responses in the experiments and the simulations, we used the model to study more systematically the impact of press disturbance duration on predator (Fig. 2a) and prey (Fig. 2b), as well as pulse disturbance strength on predator (Fig. 2c) and prey (Fig. 2d).

**Fig. 3:**
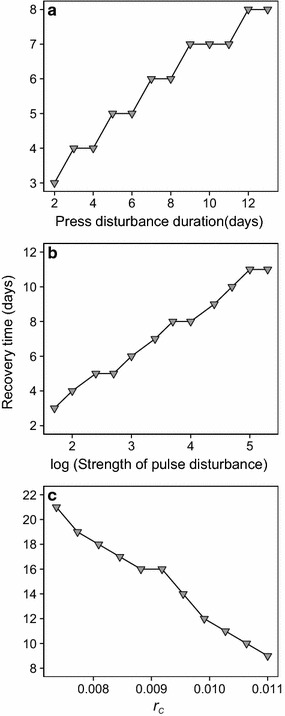
Recovery time of predator depending on the disturbance duration, strength and resource coupling. We explicitly focused on predator recovery and defined the time a predator population needed to reach the pre-disturbance population size again as “recovery time”. Recovery time is calculated as the duration (days) from the end of disturbance to the return to the pre-disturbance population size. **a** The dependence of recovery time of predator on the duration of press disturbance, **b** the strength of pulse disturbance and **c** changing resource coupling *r*_*C*_ resulting from the model simulations

The predator population declined stronger with both increasing press duration or pulse strength and recovered only slowly (Fig. 2a, c). Pulse and press disturbances resulted in a transient phase of decreased predator population sizes. With increasing disturbance impact, recovery times of the predator increased (Fig. 3a, b).

Note that, this was also valid for prey populations. However, it goes unnoticed on the daily sampling basis as the prey population recovered from disturbance within the 24 h sampling interval. Subsequently, it grew to higher population sizes, due to diminished predator stress (Fig. 2b, d). With increasing press duration and pulse strength, the chance increased that the prey population retained a high equilibrium population size for some time during or after the disturbance. Prey population size returned to the lower pre-disturbance equilibrium size only after significant recovery of the predator.

### Impact of predator coupling to an alternative resource on predator and prey transient recovery dynamics

Changes in the coupling to the alternative resource *r*_*C*_ impacted predator (Fig. 2e) and prey (Fig. 2f) dynamics considerably under pulse disturbance and in a similar way also under press disturbance (see Additional file 1: Figure S2). As expected, lower values of *r*_*C*_ resulted in lower pre-disturbance equilibrium size of the predator (Fig. 2e) and an accordingly higher prey abundance. From these levels, disturbance reduced predator abundance according to the pulse strength. In contrast, prey grew to carrying capacity (maximum size that the density dependent prey population can reach) within 24 h, independent of its pre-disturbance abundance (Fig. 2f). Recovery time of the predator extended significantly with decreasing *r*_*C*_ (Fig. 3c). Therefore, the prey population could retain its carrying capacity for a longer time (Fig. 2f).

## Discussion

We found strong impacts of the strength and duration of disturbances on the transient dynamics and recovery time of predator and prey, and strong differences among the dynamics of the two species due to their position in the food web. In particular, our results revealed a slowed recovery of the predator from the disturbance inducing a temporary phase of prey release. The predator’s coupling to an alternative resource was strongly impacting its own recovery time and thus also the length of the prey release phase. These general findings are discussed in the following in more detail.

### Transient recovery dynamics of predator and prey may result in prey release

After disturbance ceased, the predator population recovered to pre-disturbance size (Fig. 2a, c). The respective recovery time was strongly related to the disturbance duration and strength (Fig. 3a, b). This finding is highly relevant, because prolonged recovery time, during which population size is low, comes along with increased extinction risk [27]. Extinction of top predators may cause radical changes in ecosystems by altering community structures [28, 29].

We found similar structural changes in our protist-bacteria system. The prey population size considerably increased during and after the disturbances due to missing predation pressure (Fig. 2b, d). This is a clear sign of prey release [16, 30]. Effectively, disturbance had uncoupled the two interacting species, such that the prey population was no longer relevantly affected by its predator. Prey release is common in systems with substantial disturbance on predators, e.g. by hunting [31]. For example, it was previously observed that the prey population release following the hydrological disturbance in a freshwater ecosystem was due to the reduced abundance of large sized predators [16]. A similar pattern has been also observed in an island ecosystem following a hurricane which reduced the abundance of top predators and caused herbivore outbreak [15]. We found that even if disturbance is affecting both species with equal mortality, as in our study, this can initiate prey release. The duration of this prey release depended on both the duration and the strength of the disturbance (Fig. 2b, d). Thus, even if a species is heavily impacted by disturbance (such as the bacterial prey), it might still benefit due to diminished competitor or enemy stress.

### Predator coupling to an alternative resource is important for predator recovery and prey release

The use of an alternative resource is a known phenomenon for the studied protist. *Tetrahymena* species are able to grow on dissolved carbon sources and even fail to reduce the density of bacteria offered to them [32]. Foraging may be flexible due to specific predator traits such as absolute time or effort needed for grazing and relative intake rates, which, in turn impact the transient dynamics of the communities [21]. We found that a strong coupling to the alternative resource allowed the predator to reach higher pre-disturbance equilibrium sizes and accelerated the predator’s recovery after the disturbances (Fig. 2e). Accordingly, weak couplings are advantageous for the prey (Fig. 2f) and may result in prey release as well. These results support previous findings on the importance of alternative resources for food web stability [33].

### Limitations and outlook

Despite its simplicity, our simulation model well reflects the transient dynamics of both predator and prey under pulse and press disturbance. Although this simplicity greatly facilitates a general understanding of the mechanisms, it has also drawbacks coming along with some mismatches between experimental data and model results.

As explained in the results section, the experimental prey population took longer to increase than indicated by the model (Fig. 1b, c) and reached lower values after pulse disturbance (Fig. 1c). Also, the experimental predator population already started to increase, while press disturbance was still impacting the community (Fig. 1b). These responses indicated a weaker impact of disturbance on the predator than expected from the model. We therefore tested the impact of an alternative resource across a range of coupling strengths as this could attenuate the impact of disturbances on the predator. We found that coupling of the predator to an alternative resource did clearly reduce its recovery time (Fig. 3c) but could not reproduce an increase of the predator population already during press disturbance (see Additional file 1: Figure S2). Stronger consumption of an alternative resource could be possible during a phase of increased dilution rates along with very low and high prey abundance. For future work, we suggest to relax the assumption of a constant coupling and to test coupling strengths dependent on prey density.

Another mismatch is that in contrast to model projection, the experimental prey population after pulse disturbance (Fig. 1c) did not completely return to the pre-disturbance equilibrium, but remained slightly elevated. Prey adaptation mechanisms such as cell aggregation and biofilm formation may cause this deviation and might provoke an alternative system state triggered by the disturbance [34–36].

It should also be taken into account that our simple Lotka–Volterra type model ignores possible predator satiation effects (Holling Type II and Type III non-linear functional responses) and assumes a linear functional response (Holling Type I without saturation). This is because the good fit of the L–V model to the experimentally measured predator and prey growth curves (Additional file 1: Figure S1C) indicates that predator’s linear functional response describes the empirical data well and therefore density-dependent predation in form of non-linear functional responses is unlikely. However, given the discrepancies, especially during the prey release phase, one should investigate in future the applicability of non-linear functional responses. These investigations can be combined with the above described density dependent couplings to an alternative resource.

## Conclusions

By combining experimental and modelling approaches we found that the interplay of disturbance attributes and food web structure determines the transient recovery dynamics of interacting species. This can lead to diverging population growth with one trophic level suffering and the other one profiting even if disturbance induces the same mortality. Most importantly, coupling of the predator to alternative resources may stabilize the community dynamics. These findings are essential for understanding how through changing disturbance attributes or creation of alternative resources (additional couplings) the transient food web dynamics can be changed to the benefit or harm of a species. These factors should therefore be taken into account in future food web studies. Taking a closer look at the impact of disturbances on species and communities and the resulting transient recovery dynamics might turn out to be pivotal in establishing intervention tools for conservation biology, biological control and epidemiology.

## Additional file

**Additional file 1: Figure S1.** Experimental growth data and the fitted curves of prey alone, predator alone and prey–predator interaction. **Figure S2.** Impact of predator coupling *r*_*C*_ to an alternative resource besides prey under press disturbance.
