# Supplementary material for: Transient recovery dynamics of a predator–prey system under press and pulse disturbances
Source: BMC Ecol. 2017 Apr 4;17:13. doi: 10.1186/s12898-017-0123-2 (PMC5381073; doi:10.1186/s12898-017-0123-2)
Supplement: Supplementary file 1 — Additional file 1: Figure S1. Experimental growth data and the fitted curves of prey alone, predator alone and prey–predator interaction. Figure S2. Impact of predator coupling r C to an alternative resource besides prey under press disturbance. [file 12898_2017_123_MOESM1_ESM.docx]

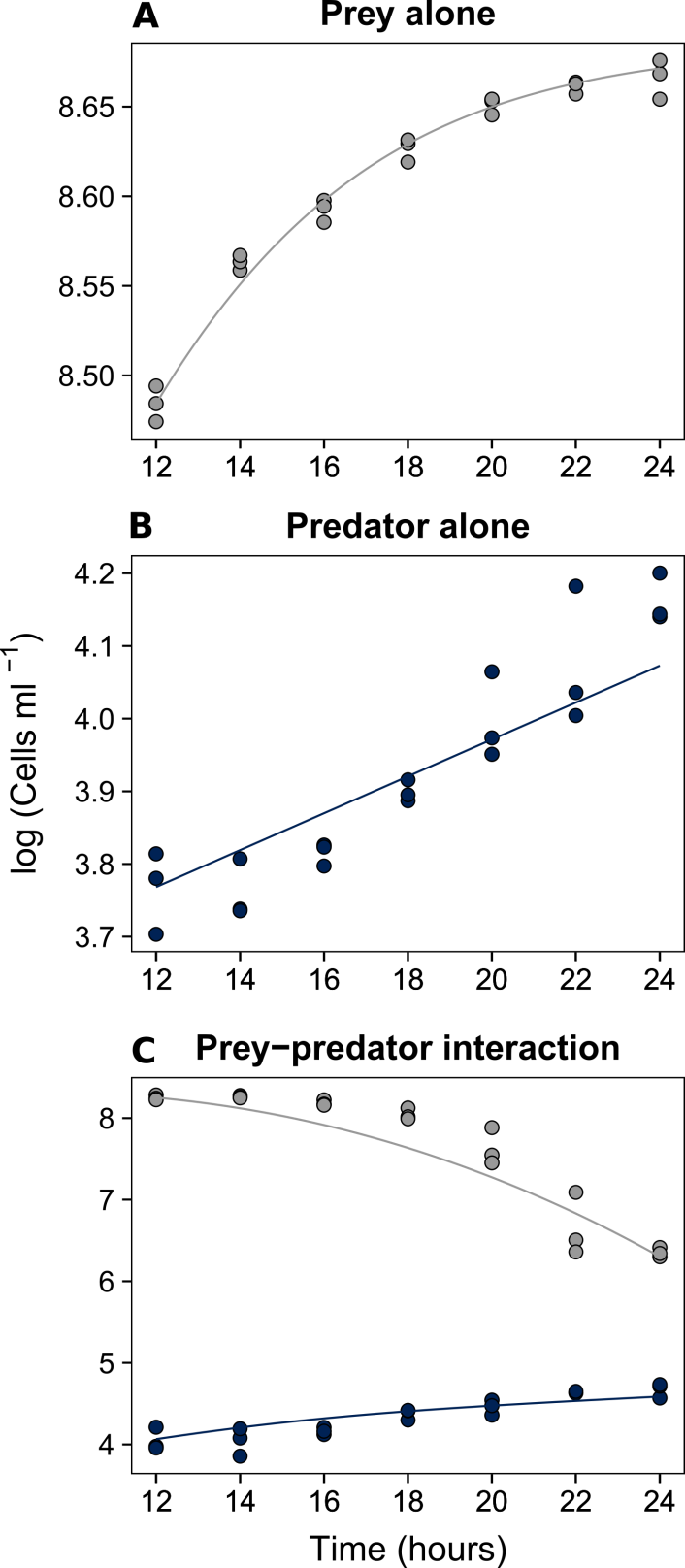
**Additional file 1**

**Figure S1:** Experimental growth data (circles) and the fitted curves (solid line) of prey alone (A), predator alone (B) and prey-predator interaction (C) as described in the methods section of the main text.


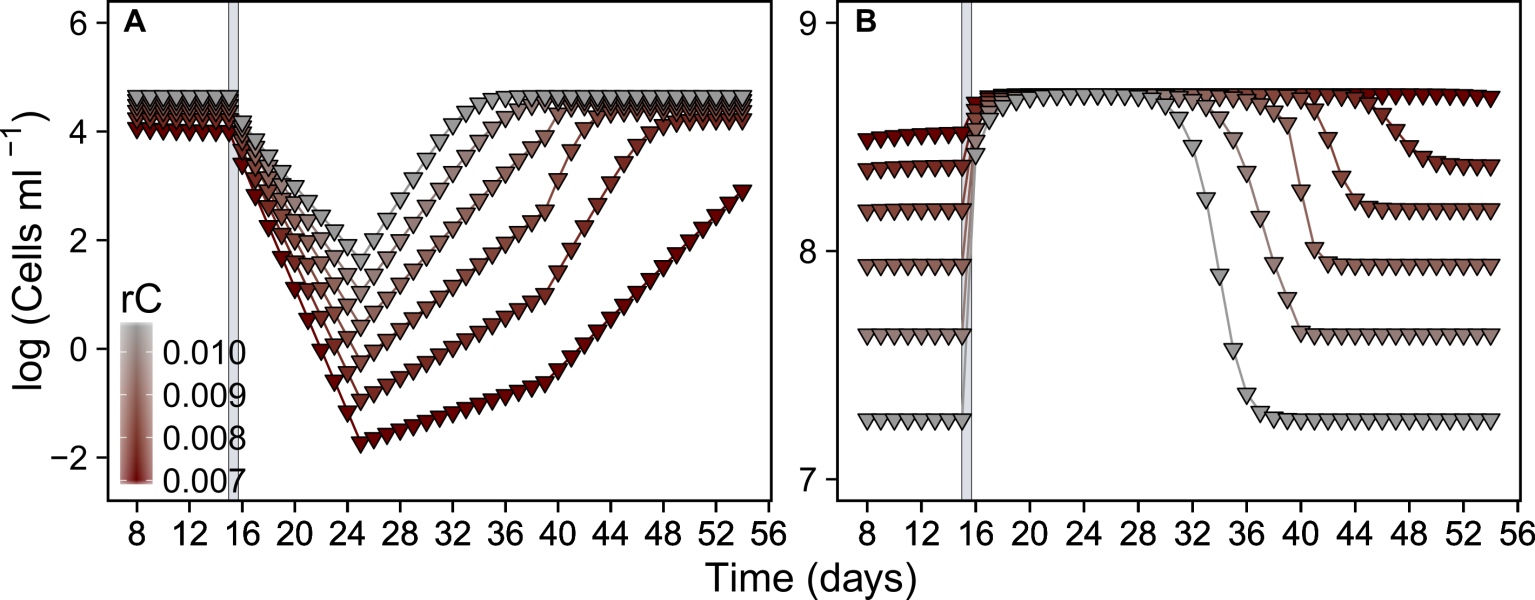


**Figure S2:** Impact of predator coupling “*r_C_*” to an alternative resource besides prey (varied from 0.007 to 0.011) under press disturbance (40 fold dilution) projected by the model simulations for predator (A) and prey (B). Color gradient shows lowest (grey) to highest (dark red) *r_C_* values. Disturbance action is shown as grey shadows.
